# Lexical Leveraging in Novel Word Learning: Different Semantic Properties Support Learners at Different Stages of Development

**DOI:** 10.1111/desc.70087

**Published:** 2025-11-04

**Authors:** Amanda Rose Yuile, Justin B. Kueser, Claney Outzen, Sharon Christ, Risa Stiegler, MaryCarson Adams, Barbara Brown, Arielle Borovsky

**Affiliations:** ^1^ Purdue University West Lafayette Indiana USA; ^2^ Boys Town National Research Hospital Boys Town Nebraska USA

**Keywords:** perceptual information, semantic features, taxonomic structure, vocabulary development, word learning

## Abstract

Toddlers better retain novel object‐label mappings from taxonomic categories they have more knowledge of. Separately, words for concepts with more perceptual features are learned earlier than words for concepts with fewer perceptual features. Because these factors have only been examined separately, it is unclear whether the effects of taxonomic density stem from differences in structured taxonomic knowledge or simply reflect lower‐level differences in perceptual similarity among concepts. We asked how taxonomic structure and perceptual information jointly contribute to word learning at 24 months old in an ostensive word learning task. We found that semantic category knowledge facilitated word learning. We also found that the availability of perceptual features served as additional supports for word learning by children with smaller expressive vocabularies. This indicates that structured taxonomic knowledge is a better predictor of word learning compared to lower‐level perceptual features at 24 months old. However, perceptual cues may provide additional support for vocabulary growth at the start of development.

**Summary:**

- We explore how semantic category knowledge and perceptual features jointly influence novel word learning at 24 months old in an ostensive word learning context.
- Novel word learning was facilitated within semantic categories the toddlers knew more about, when controlling for the availability of perceptual information.
- Toddlers with smaller productive vocabularies used perceptual features as additional supports for word learning, but those with larger vocabularies did not.
- These findings show that structured taxonomic knowledge is a better predictor of word learning at 24 months old compared to lower‐level perceptual information.

## 1 Introduction

To what extent do learners leverage their existing semantic knowledge to support word learning? A growing body of evidence suggests that children learn words more accurately and deeply in categories about which they have a broader or denser basis of knowledge (e.g., Borovsky 2020; Borovsky et al. 2016a; Hadley et al. 2019). In the current paper, we seek to enrich this account by asking: *What semantic information specifically are children leveraging?*

**FIGURE 1:**
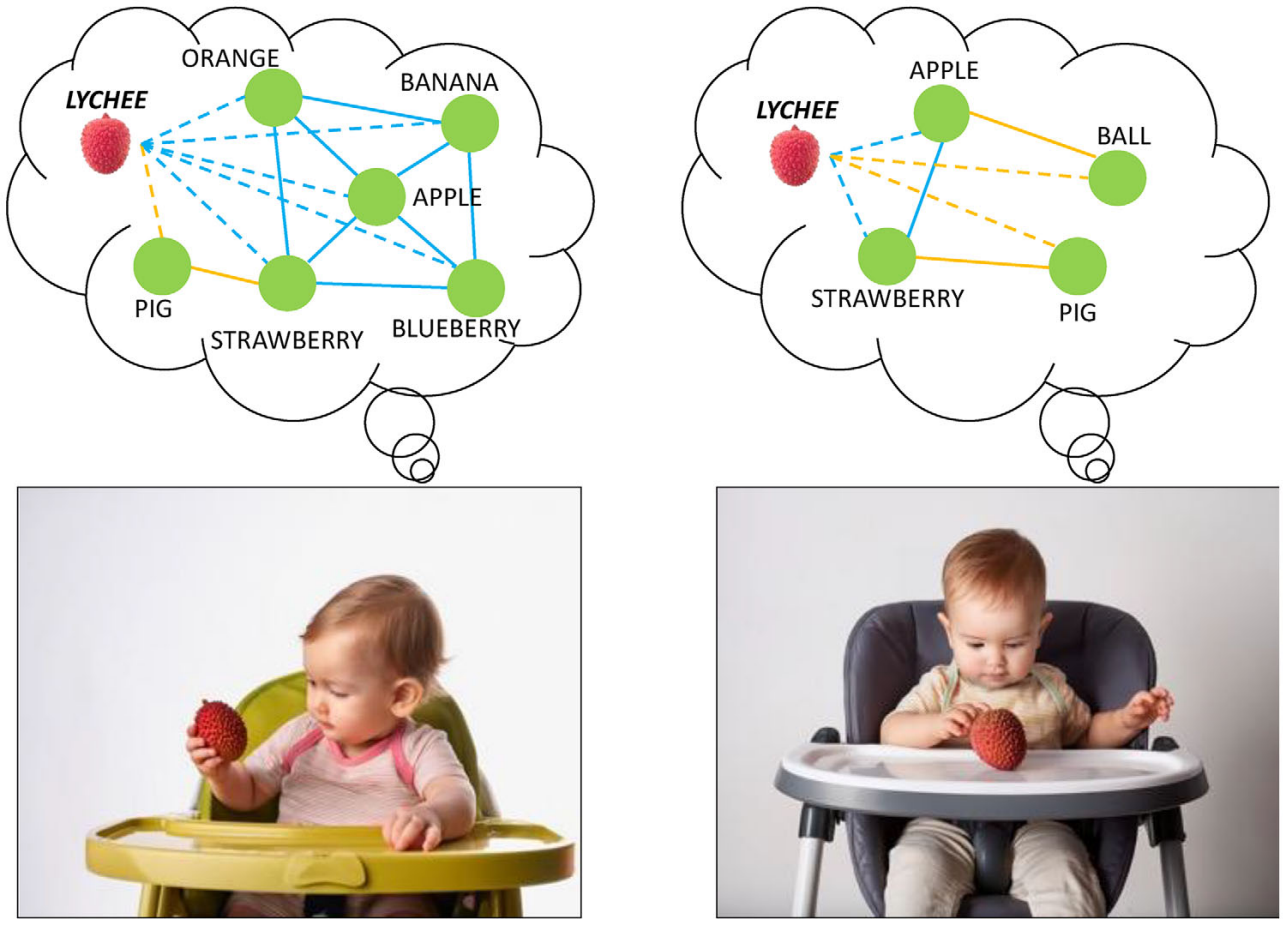
Two toddlers sitting in highchairs looking at a lychee. Thought bubbles over each toddler contain toy semantic networks where nodes (circles) represent concepts and edges (lines connecting circles) represent shared meaning. Solid lines represent connections between existing semantic knowledge. Dashed lines represent potential relationships between the novel concept *lychee* and the toddler's existing knowledge. Blue lines represent shared taxonomic meaning, and yellow lines represent shared perceptual meaning. Images were generated using Adobe Firefly.

Adult lexicons encode relations between word meanings among multiple dimensions, such as taxonomic similarity (i.e., membership in hierarchical categories) and perceptual similarity (i.e., features that are accessible to the senses). Each of these semantic dimensions may have differing consequences for learning. To illustrate, consider a situation, as in Figure 1, where two toddlers with different knowledge regarding fruit, encounter a new fruit, *lychee*. Semantic leveraging accounts posit that when encountering this new concept, the toddler who knows many other fruits may more readily infer that this object is edible and tastes sweet without having eaten the object if they recognize its similarity to the other fruits that they know. In contrast, the toddler with sparser category knowledge may have more difficulty making this generalization, having not yet acquired enough related concepts to identify the new item as a fruit, with all the category's attendant features. However, taxonomic category membership represents only one dimension along which we may organize concepts in our semantic memory. In Figure 1, some concepts are related via shared taxonomic information (e.g., both apple and banana are fruits), while others are related via shared perceptual characteristics (e.g., both apple and ball are round). Each of these dimensions of meaning might separately contribute to early vocabulary growth. For example, while the toddler who knows more fruits may know more about the *lychee* for free, the toddler who knows fewer fruits may also make inferences about the new item—for example, that *lychee* will roll across the floor if dropped, based on the perceptual feature <*is_round*>, which is shared between lychee, apple, and ball in their semantic network. Thus, each of these two dimensions of meaning support the generation of inductive inferences, albeit about different characteristics of the new object.

We further investigate the semantic leveraging account of early language learning by exploring how different types of semantic relations between words support learning. Specifically, we ask how **semantic category knowledge** and **perceptual features of concepts** jointly contribute to novel word learning in 24‐month‐olds with wide variation in language skills. Implicit in the argument we outline below is the notion that word learning is inherently tied to the structure or organization of our existing *conceptual* representations. That is, we assume that learning a new word involves integrating the word into existing conceptual structures, and that semantic knowledge provides a way of storing or expressing conceptual knowledge using language. As such, these two processes (word learning and conceptual development) support each other.^1^ In the current paper, we consider how existing semantic knowledge, focusing on taxonomic and perceptual meanings, influences the relative robustness of word‐referent associations formed within unambiguous learning conditions (i.e., in an ostensive labeling context).

Semantic category knowledge is linked to word learning in toddlers in a variety of ways. For example, toddlers' ability to spontaneously sort objects into categories is linked to the onset of the vocabulary spurt (Gopnik et al. 1996; Gopnik and Meltzoff 1992; Poulin‐Dubois et al. 1995). This connection suggests that toddlers’ ability to impose hierarchical organization on concepts facilitates learning of new words/concepts. More directly, recent research demonstrates advantages in language processing and word learning in semantic domains for which children have relatively more knowledge. For instance, Borovsky et al. (2016a) found that 24‐month‐olds were faster to fixate on familiar targets from domains about which they had relatively denser versus sparser knowledge and showed more robust anticipatory fixations on thematically appropriate direct objects from denser semantic domains (e.g., looking to a car after hearing “you can drive the…”). Further, novel word learning is facilitated in relatively denser semantic categories (Borovsky et al. 2016a). That is, following ostensive labeling, 24‐month‐olds looked more robustly toward novel targets (low‐frequency items from early acquired categories, e.g., *lychee*) that came from categories about which they had more (vs. less) knowledge. Finally, semantic category knowledge helps children deal with challenging word‐learning conditions. That is, when words are taught in conditions that introduce ambiguity in the word‐referent mapping (i.e., in a mutual exclusivity task), only words with dense category knowledge show evidence of learning (Borovsky 2020). This latter finding implies that semantic category knowledge can ease the process of referential ambiguity resolution.

Perceptual elements of meaning can also support early word recognition and learning. For example, early learned words tend to be concrete, imageable, and have salient perceptual features (Caselli and Pyers 2017a; Rosa et al. 2010a; Gilhooly and Logie 1980a; Hansen 2017a; McDonough et al. 2011; Perry et al. 2017; Pruden et al. 2006). Children also attend to perceptual regularities across objects (Rakison, 2003). For example, the shape bias—a tendency for learners to extend new labels to objects that match the shape of the initial exemplar (Landau et al. 1988)—is one well‐documented perceptual advantage in word learning. Attentional learning accounts suggest the shape bias emerges through a process by which children learn to attend to perceptual features that provide informative and reliable cues to word meaning, with shape being one (but not the only) informative perceptual cue (Colunga and Smith 2005; Smith et al. 1996, 2002; Smith and Samuelson 2006). Toddlers also learn words earlier when they have more perceptual features in common with words that they already know (Kueser and Borovsky 2024; Peters and Borovsky 2019). Known words with more perceptually‐based feature connections to other known words are recognized more accurately in online comprehension than known words with fewer perceptually‐based connections (Peters et al. 2021). Finally, words with meanings that involve more distinct sensory modalities are learned earlier according to parental vocabulary checklists and are recognized more quickly in a looking‐while‐listening task (Seidl et al. 2024). Thus, the availability of perceptual features of concepts supports early word learning and semantic representation.

Taxonomic structure and perceptual features are correlated. For instance, all mammals have hair/fur (a defining perceptual characteristic of category membership), and all mammals have mammary glands to feed their offspring (a defining functional characteristic of category membership). As adults, we may view the biological trait <*has_mammary_glands*> as more central in organizing the mammal category relative to <*has_hair>*—a hairless cat is a mammal, and a fuzzy caterpillar is not. However, the co‐occurrence of perceptual features among category members may provide a reasonable early superordinate category representation. This may even be the case when non‐observable and surface‐level feature overlap among category members is not absolute. For instance, most adults consider the functional feature *<can_fly>* to be central in defining the bird category, but readily accept non‐flying category members, such as penguins. In more formal terms, even a correlation of less than 1 can still carry meaningful information about the structure of the world and guide learning.

The idea that perceptual feature overlap among categories members provides a reasonable early category representation is supported by empirical and theoretical work. Models of lexical organization constructed with perceptual features of early learned nouns resemble the hierarchical taxonomic structure of adult lexical networks (Hills et al. 2009a), suggesting a strong correlation between these two dimensions of meaning. Therefore, it is possible that what has been construed as taxonomic structure effects in prior work were driven by perceptual features. This possibility is consistent with theoretical accounts which suggest that children prioritize perceptual information in early word learning. According to the *perceptual‐to‐conceptual shift* (see e.g., Quinn and Eimas 1997), children's semantic knowledge is initially organized by perceptual relations among early learned concepts. Taxonomic structure later emerges as a secondary reorganization of first‐order perceptual information.

However, alternative theories of early conceptual development, such as the *thematic‐to‐taxonomic shift* (see e.g., Smiley and Brown 1979) propose that abstract semantic information, which supports exclusive categorization, is available from the start of development. This latter possibility is supported by work demonstrating that taxonomic organization provides better discrimination between categories compared to perceptual organization only (Hills et al. 2009a), and that taxonomic features of early acquired words relate to the order that words are learned, though to a lesser degree than perceptual features (Peters and Borovsky 2019).

One potential avenue for discriminating between these two theoretical perspectives is to contrast taxonomic and perceptual information with respect to overall vocabulary development. One clear hypothesis which follows from the perceptual‐to‐conceptual shift is that access to higher‐order taxonomic features of concepts depends on the acquisition of conceptual knowledge. Under this hypothesis, children's higher‐density taxonomic categories are likely to have undergone a process of reorganization, supplementing what had been solely perceptual associations among category members with higher‐level taxonomic structure. Thus, effects of taxonomic density or perceptual features may interact with vocabulary size to predict learning; children may come to rely more on taxonomic structure and less on perceptual features as their vocabularies grow. We note that to truly test this hypothesis, one needs longitudinal data. The current paper, therefore, represents an initial foray into this question, and we return to this concern in the Discussion section.

Several findings suggest that sensitivity to taxonomic information increases as a function of vocabulary size. In semantic priming studies, 24‐month‐old children reliably show a priming effect when the prime and the target are taxonomically and/or associatively related, while findings with 21‐month‐olds vary, sometimes showing a priming effect and sometimes not (Arias‐Trejo, and Plunkett, 2009 2009). Additionally, novel word learning is associated with vocabulary skill. For instance, Bion et al. (2013) found that 24‐month‐olds with larger vocabularies were better able to map a novel label to its intended referent in a mutual exclusivity task. And Borovsky et al. (2016a) found that children with larger vocabularies reliably learned a novel word from both higher and lower density taxonomic categories, while children with smaller vocabularies only showed evidence of learning in higher‐density domains. Thus, the influence of taxonomic information in word learning may vary with vocabulary size.

### 1.1 The Current Study

Because the effects of taxonomic and perceptual information on word learning have only been examined separately, it is unclear whether the effects of taxonomic density stem from differences in structured taxonomic knowledge or simply reflect lower‐level differences in perceptual overlap between concepts. We seek to understand how taxonomic category density and perceptual richness may jointly contribute to word learning by manipulating both properties in a word learning task. The current study design is, in part, based on Borovsky et al. (2016a), which, to remind readers, found that 24‐month‐olds’ word learning was facilitated for categories that were higher (vs. lower) in density according to the child's own vocabulary. This study expands on this finding by asking whether perceptual richness affects novel word learning and how this factor interacts with taxonomic category density. To address these questions, we measured 24‐month‐olds’ retention of novel object label mappings following an ostensive word learning period. While much of early word learning happens in ambiguous learning environments that require the child to generate inferences about word meanings (see Gleitman and Trueswell 2020), we focus here on learning within unambiguous contexts. The reason for this is to present a simplified version of the learning problem for an initial foray into disentangling the joint contributions of taxonomic and perceptual dimensions of meaning. We used a 2 × 2 within‐subjects design to explore how higher and lower taxonomic category density and higher and lower perceptual richness contribute individually and in combination. We additionally ask how these factors interact with toddlers’ current vocabulary size to predict word learning, as a general proxy for developmental progress as outlined above.

#### 1.1.1 Measuring Taxonomic and Perceptual Structure

We adopt a semantic feature‐based system for classifying word meaning developed by Cree and McRae (2003), motivated by theories of lexicosemantic representation. For example, the word CAT may have the features <*has_fur>*, <*is_an_animal>*, <*has_four_legs>*, etc. These features can be empirically derived (see McRae et al. 2005 and Borovsky et al. 2023 for details). These features can also be classified into broad types (as proposed by Cree and McRae 2003): taxonomic, perceptual, functional, and encyclopedic (see Table 1 for definitions). This approach to semantic representation has been used to investigate how lexical items are related and represented in memory (e.g., Cree et al. 2006; Grondin et al. 2009; McNorgan et al. 2007) and has been extended to questions concerning acquisition (e.g., Hills et al. 2009a, 2009b; Kueser et al. 2023; Peters and Borovsky 2019; Peters et al. 2021).

**TABLE 1:** Semantic feature types, definitions, and examples based on work from Cree and McRae (2003) as well as Borovsky et al. (2023).

Feature type	Definition	Examples (concept = CAT)
Taxonomic	Information about position in a hierarchical category	*<is_an_animal>* *<is_a_pet>*
Perceptual	Information immediately accessible via the senses	*<has_fur>* *<has_four_legs>*
Functional	Information about object interactions	*<eats_food>*
Encyclopedic	Information that does not fit into the other three feature type categories	*<is_nice>*

#### 1.1.2 Predictions

We expected children's success in mapping novel words to their intended referents would be associated with their looking behavior, such that successful mapping would result in greater looking toward the target relative to the distractor image during test trials. We also expected differences in mapping behavior across conditions, outlined below.

##### 1.1.2.1 Hypothesized Effects of Taxonomic and Perceptual Information

Based on previous findings (Borovsky et al. 2016a), we expected taxonomic information to facilitate word learning such that children would look more to the target if the target novel word belonged to a higher‐density compared to lower‐density category.

Prior work demonstrates that words’ number of perceptual features is negatively associated with age of acquisition, and known words with more perceptual features are recognized more accurately in online comprehension than words with fewer perceptual features (Peters and Borovsky 2019; Peters et al. 2021). On this basis, we hypothesized an effect of perceptual richness such that children would look more at targets with more perceptual features than targets with fewer perceptual features.

##### 1.1.2.2 Hypothesized Interaction of Taxonomic and Perceptual Information

The key question concerned how word learning would be influenced by the joint contribution of taxonomic and perceptual information. Here, we outlined two possibilities: (1) If taxonomic and perceptual information interact, we expected a larger effect of perceptual richness in lower‐density (vs. higher‐density) categories. This hypothesis is generally aligned with theoretical accounts of conceptual development (e.g., Quinn and Eimas 1997) that propose taxonomic structure emerges as a secondary reorganization of first‐order perceptual information. (2) If taxonomic and perceptual information do not interact, we expected children to use all available cues without giving priority to either source of information.

##### 1.1.2.3 Hypothesized Effects of Vocabulary Size on Novel Word Learning

A final factor we considered was vocabulary size. We expected children with larger vocabularies to look more to the target (relative to the distractor) during test trials, as in prior work. We also hypothesized interactions between vocabulary size, perceptual richness, and taxonomic density. Specifically, we predicted an interaction of vocabulary size and category density such that children with larger vocabularies would show increased effects of category density because accessibility of taxonomic information is related to vocabulary size (Chow et al. 2019; Rämä et al. 2013). We also predicted an interaction of vocabulary size and perceptual richness such that children with larger vocabulary sizes would show decreased effects of perceptual richness, indicating a decreasing reliance on perceptual information as vocabularies grow.

## 2 Methods

### 2.1 Participants

This experiment was conducted as a part of a larger, longitudinal research project for which 160 families were enrolled. Of these families, 135 twenty‐four‐month‐old children (*M* age = 24.4 months, range = 23.7–25.9 months) contributed data to the analyses. See Table 2 for sample demographics.

**TABLE 2:** Demographic characteristics of participants.

Characteristic	*n*	%
Sex		
Male	71	52.6%
Female	63	46.7%
Prefer not to say	1	0.7%
Maternal education		
High School Graduate	3	2.2%
Some College, No Degree	10	7.4%
Associate Degree	7	5.2%
Bachelor's Degree	49	36.3%
Master's Degree	47	34.8%
Professional School Degree	8	5.9%
Doctoral Degree	11	8.1%
Race		
American Indian/Alaska Native	1	0.7%
Asian	2	1.5%
Black or African American	7	5.2%
Multiracial	14	10.4%
Other	1	0.7%
White	110	81.5%
Ethnicity		
Hispanic or Latino	2	1.5%
Not Hispanic or Latino	132	97.8%
Prefer not to say	1	0.7%

Participant exclusions were as follows: 12 families did not participate in this timepoint of the longitudinal project, two toddlers were born prematurely, two toddlers did not pass a hearing screening, one toddler demonstrated atypical cognitive development by scoring less than 77.5 (standard score) on the Cognitive subtest of the Bayley Scales of Infant Development: 4th Edition (Bayley and Aylward 2019) administered at 18 months, one toddler demonstrated symptoms of Autism Spectrum Disorder by scoring greater than 30 (standard score) on the Child Autism Rating Scale (Schopler and Van Bourgondien 2010) administered at 24 months, one toddler did not attempt eye tracking, and six toddlers were removed for failure to contribute enough trials following data cleaning procedures (see Data Processing).

Families were paid $30 an hour and toddlers were given a children's book at each of 2 h‐long visits. The experimental hypotheses and planned analyses were preregistered at https://osf.io/5jwcu/.

### 2.2 Materials

#### 2.2.1 Item Selection

We systematically manipulated taxonomic density and perceptual richness within subjects. We used a 2 × 2 design in which each factor (category density and perceptual richness) had two levels (higher or lower). We identified four taxonomic categories that are familiar to toddlers and were used in the prior experiment with a similar design (Borovsky et al. 2016a): animals, drinks, fruits, and vehicles. We focus on concrete, basic‐level concepts/labels from categories that are all highly imageable and familiar to toddlers. In our initial design of the experiment, we relied on our own intuitions regarding imageability. However, based on constructive feedback from a reviewer, we searched for each category congruent MBCDI word within a set of imageability ratings from Coffey et al. (2024). We observed that each category had an average imageability rating higher than 6 on a 7‐point Likert scale. These high imageability ratings provide empirical support that the categories tested were all highly imageable.

Category density was assigned based on each individual toddler's existing vocabulary knowledge (see Analytic Plan). We chose two unfamiliar words in each category so each category had one item with low perceptual richness and one item with high perceptual richness. Thus, there was a total of eight novel items, two per category across four categories (see Table 3).

**TABLE 3:** Novel items.

			Perceptual feature		
Category	Label	Image	Category	Count	List
Animals	Hedgehog (hɛʤhɒɡ)	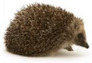	Low	6	*<is_small>* *<is_brown>* *<has_a_snout>* *<has_eyes>* *<has_a_nose>* *<has_claws>*
	Tapir (teɪpɪr)	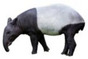	High	9	*<has_legs>* *<is_white>* *<has_4_legs>* *<is_black>* *<has_a_snout>* *<has_a_nose>* *<has_a_tail>* *<is_large>* *<has_ears>*
Drinks	Boba (bobʌ)	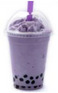	Low	4	*<is_purple>* *<is_cold>* *<tastes_sweet>* *<tastes_good>*
	Falood (falud)	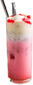	High	10	*<tastes_sweet>* *<is_pink>* *<is_white>* *<is_cold>* *<is_colorful>* *<fruit_flavoured>* *<is_creamy>* *<is_red>* *<has_toppings>* *<tastes_good>*
Fruit	Mamey (mʌmeɪ)	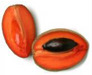	Low	5	*<has_seeds>* *<is_orange>* *<is_brown>* *<tastes_sweet>* *<is_red>*
	Lychee (laɪ⁠tʃi)	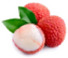	High	7	*<tastes_sweet>* *<is_red>* *<is_white>* *<has_leaves>* *<is_small>* *<is_soft>* *<has_green_leaves>*
Vehicles	Draisine (dɹeɪzin)	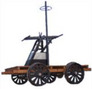	Low	3	*<has_wheels>* *<has_4_wheels>* *<is_brown>*
	Gokart (ɡoʊkɑrt)	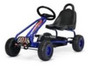	High	8	*<is_fast>* *<has_wheels>* *<has_a_seat>* *<has_4_wheels>* *<has_a_steering_wheel>* *<has_tires>* *<is_blue>* *<is_small>*

Perceptual richness was defined as the raw count of perceptual features for each item in a database of semantic features. To collect this database, we recruited 30 adult native English speakers from Prolific to describe the semantic characteristics associated with each novel item. Semantic features that were mentioned by at least 16.66% (5 of 30) of participants were subject to an additional round of norming in which the semantic features themselves were rated by a second set of 30 adult native English speakers on Prolific. In this second round, participants were asked to rate how accessible each *feature* is to toddlers on a scale of 1 (very unlikely to be a part of toddlers’ knowledge) to 7 (very likely to be a part of toddlers’ knowledge). It would not be developmentally appropriate to ask toddlers to produce lists of semantic features for each novel concept. However, the features that an adult knows may not necessarily be accessible to toddlers. Thus, we ask for adult ratings of toddler‐accessibility in an effort to only include features that toddlers are likely to have access to. Some existing work suggests that adults underestimate children's knowledge (citations), thus, it is likely that these toddler‐accessibility ratings offer a conservative estimate of what children know. Features that received average toddler accessibility ratings of less than 4 were discarded. Final counts of features for each experimental item are listed in Table 3. Please see Borovsky et al. (2023) for a more detailed description of the feature norming procedures. Importantly, the items we chose, while infrequent, were not atypical category exemplars as illustrated by the list of perceptual features adults produced for each (see Table 3).

#### 2.2.2 Visual Stimuli

Novel items were depicted using photorealistic images that were 400 × 400 pixels in size and centered on a white background. We chose 100 × 100‐pixel images to serve as central fixation targets for drift correction (see Eye Movement Recording) and gaze‐contingent central fixations within the experimental trials (see Procedure).

#### 2.2.3 Audio Stimuli

All audio stimuli were recorded in infant‐directed speech by a female native speaker of General American English at 48 kHz and normalized to a mean intensity of 70 dB. Ostensive labels for training trials were adjusted to a duration of 12,466 ms during which the novel label was repeated five times. Target object labels for test trials were adjusted to a standard duration of 660 ms. We also recorded encouraging phrases to help maintain toddlers’ interest and engagement in the task. The phrases “Look!” and “Ooh!” were recorded to direct toddlers’ attention to central fixations and phrases such as “Do you like it?” and “Wow, that's fun!” were recorded to be played after the label during all test trials.

### 2.3 Procedure

All assessments were completed across two laboratory visits that lasted 1 h each. We aimed to schedule the second visit within 14 days of the first (average number of days between visits = 8.5, range = 0–35^2^).

#### 2.3.1 Eye Movement Recording

Toddlers were seated in front of a computer monitor. A caregiver and an experimenter (E1) sat on either side of the child. Caregivers wore noise cancelling headphones with instrumental music playing so they would not hear the novel labels. E1 directed the child's attention to the screen as needed. A second experimenter (E2) sat behind the computer monitor (hidden by a curtain) and operated the eye tracker. At the start of the experiment, E1 told the child that they would watch some movies and look at some pictures.

Eye movements were recorded with a SR Research EyeLink 1000+ eye tracker in a remote arm configuration at a sampling rate of 500 Hz^3^. Stimuli were presented using SR Research's Experiment Builder software. Before the experiment began, E2 performed a manual 5‐point calibration and validation procedure using an animated calibration target. Before each trial of the experiment, a “drift check” procedure was performed^4^.

#### 2.3.2 Novel Word Training and Test

**FIGURE 2:**
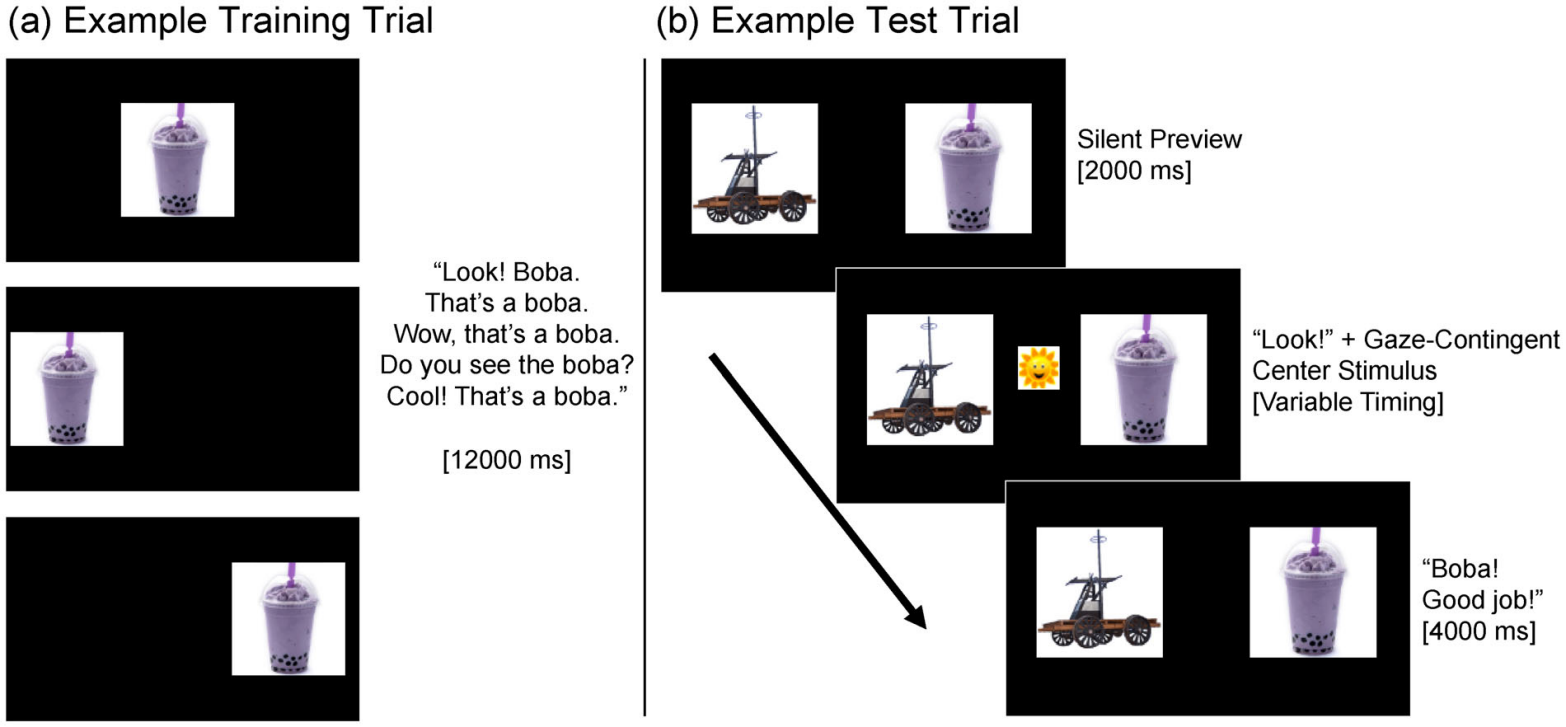
Example training (a) and test (b) trials.

The design contained four experimental blocks and 32 total trials. Novel items appeared as yoked pairs with one pair per block. Items were paired such that within pairs, the items came from different categories, and one item had higher perceptual richness and one had lower perceptual richness. Each block began with four training trials (two per word) in which the novel object moved back and forth across the screen while the novel label played five times, for a total of 10 labeling instances per novel item. This was followed by four test trials (two per novel word) in which both items were displayed, and one was labeled. Each test trial began with a 2000 ms silent visual preview of the images. Next, a gaze‐contingent central fixation appeared with an auditory cue (“Look!” or “Ooh!”) and remained on screen until the toddler fixated the image for 150 ms. Finally, the label of the target novel object played (e.g., “Lychee!”). An encouragement phrase was played 2000 ms after label onset (e.g., “Good job!”). The trial ended 4000 ms after label onset; eye movements were recorded from the beginning of the preview period through the end of the trial. See Figure 2 for trial examples. Test trials were separated by 1–3 filler trials with familiar objects and words, which were included as part of another experiment.

The experiment was counterbalanced such that target objects appeared equally on the left and right sides of the screen during test trials within and across lists, and the target/distractor objects were labeled an equal number of times. We additionally counterbalanced the stimuli to account for the order in which the words were presented during training and testing. This resulted in a total of 24 counterbalance lists. The eye‐tracking task was split across visit days; toddlers completed half of the task on the first visit and half on the second visit.

#### 2.3.3 Vocabulary Assessment

**FIGURE 3:**
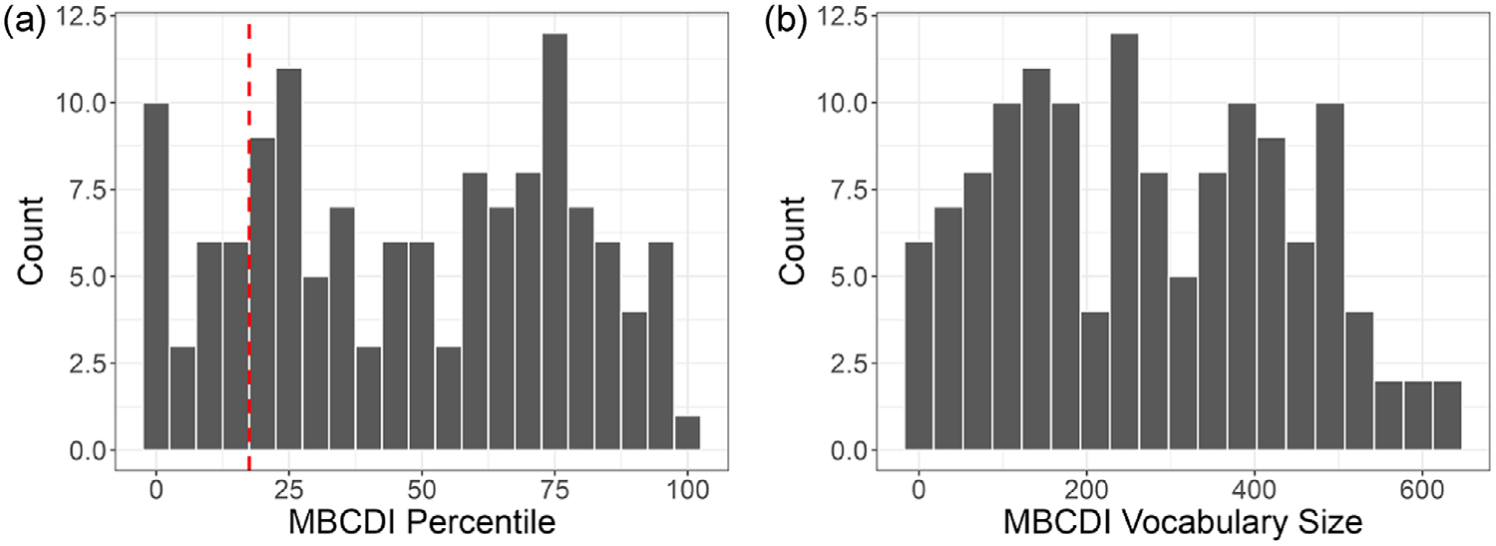
Distribution of MBCDI (a) percentile scores and (b) words produced. The red line in (a) shows the 20th percentile, a criterion for identifying children with late‐talking versus typical development.

One week prior to their first visit, caregivers were asked to complete the MacArthur Bates Communicative Development Inventory: Words and Sentences (MBCDI; Fenson et al., 2007) as a measure of their child's expressive vocabulary composition. If caregivers completed the MBCDI more than 3 days before their first visit, they were asked to update it in the lab. Vocabulary percentiles (Figure 3a) in this sample ranged from the 1st to the 99th percentile (*M* = 47.40, *Mdn* = 50.00, *SD* = 29.10). Toddlers’ vocabulary sizes (Figure 3b) ranged from 1 to 639 words (*M* = 271.10, *Mdn* = 261.00, *SD* = 167.85).

#### 2.3.4 Knowledge of Experimental Stimuli

At the end of the second visit, caregivers completed a post‐experiment vocabulary checklist. On this checklist, caregivers rated whether their child had each novel word in their productive vocabulary as 1 (Child doesn't say), 2 (Fairly sure child doesn't say), 3 (Fairly sure child says), 4 (Child says), or DK (Don't know if child says). Experimental items marked 3 or 4 were removed from the analyses (*N* trials = 40, 2% of total trials).

### 2.4 Approach to Analysis

#### 2.4.1 Data Processing

We binned target and distractor looking proportions into 50 ms bins. In training trials, we defined a single interest area (IA) which encompassed the image's entire path of movement. Our pre‐registered plan was to remove test trials that corresponded to novel items, which children failed to attend to a minimum of 20% of one training trial. Toddlers were highly attentive to training trials; no trials were removed.

We calculated the proportion of track‐loss that occurred in test trials starting at label onset and ending 4000 ms after label onset. We defined two IAs in the test trials corresponding to the target and distractor images and excluded trials in which participants failed to attend to either of the IAs for at least 20% of this period (*N* = 103 trials, 5% of trials). Next, we removed participants who did not retain at least two trials per condition (*N* = 6 participants). The final sample includes 135 participants and 1876 trials.

We calculated log‐adjusted target advantage scores (i.e., log gaze) in each bin. Log gaze was calculated as the natural log of the proportion of looking to the target over the proportion of looking to the distractor (ln[target_prop/distractor_prop]). Because values of 0 are undefined by this formula, we replaced 0 values with a value corresponding to half the value of the smallest observable nonzero target or distractor looking proportions at 500 Hz before performing the log adjustment. This log gaze procedure follows prior work (e.g., Borovsky 2020). We use log gaze because it is not limited to values between 0 and 1, making it a more appropriate outcome measure for linear regression analyses.

#### 2.4.2 Deciding on a Time Window for Analysis

Because word recognition effects are unlikely to persist across the entire 4000 ms trial duration, averaging across this window is likely to mask effects of interest. Familiar word recognition effects are usually found within the first 2000 ms after label onset (Fernald et al. 2008, while some evidence suggests novel word learning effects may emerge later (Bion et al. 2013; Borovsky et al. 2016a). We chose a data‐driven approach to determine a time window for analysis^5^: We calculated a moving average of log gaze for 1500 ms segments starting at label onset and ending 4000 ms after label onset, and chose the window with the largest average log gaze for further analysis. Importantly, we collapsed across experimental conditions to determine at which time looks to targets most exceeded looks to distractors. The resulting data‐driven analysis window is a period from 1000 to 2500 ms after label onset. This approach has the advantage of allowing us to control for the duration of the analysis window, supporting direct comparison with windows commonly used in prior research. We directly compare this data‐driven approach to a standard time window from 500 to 2000 ms after label onset. After direct comparison, we interpret the results from the best‐performing model.

#### 2.4.3 Statistical Modeling

We used a linear mixed‐effects regression to model average log gaze in our analysis windows. We included the fixed effects of category density, perceptual richness, and their interaction. We additionally included vocabulary size (count of MBCDI words produced) as a predictor, as well as the interactions of vocabulary size and density or richness. We included maternal education and participant sex as covariates in an initial model, but dropped sex from the model as it did not account for significant variance in log gaze. We included random intercepts of participant and item, and the random slope of category density by participant. Our final model, after removing nonsignificant covariates and random slopes with near‐zero variance was:

$$\begin{array}{ccc} & & \mathrm{Log}\_\mathrm{gaze}∼\mathrm{Category}\_\mathrm{density}*\mathrm{Perceptual}\_\mathrm{richness}\\ & & \;+\;\mathrm{Vocabulary}\_\mathrm{size}*(\mathrm{Category}\_\mathrm{density}\\ & & \;+\;\mathrm{Perceptual}\_\mathrm{richness})+\mathrm{Maternal}\_\mathrm{education}+\left(1|\mathrm{Item}\right)\\ & & \;+\;\left(\mathrm{Category}\_\mathrm{density}|\mathrm{Participant}\right) \end{array}$$

Model structure was determined using the data‐driven analysis window and an identical model was run for the standard analysis window.

We used effects coding for categorical predictors. We centered and scaled all continuous predictors. We used R version 4.2.2 (R Development Core Team 2008), *lme4* version 1.1–35.1 (Bates et al. 2015), and *emmeans* version 1.9 (Lenth 2019).

#### 2.4.4 Assignment of Higher and Lower Category Domains

We followed procedures outlined in Borovsky et al. (2016a) to assign higher‐ and lower‐category density within subjects. For each child, we used their MBCDI report to first calculate the proportion of words within each category (animals, fruits, drinks, and vehicles) that the child said out of the total number of words in the category. The two categories with the highest proportions were assigned to higher density, and the two categories with the lowest proportions were assigned to lower density. There were seven participants with ties between the second‐ and third‐ranked category proportion; we chose to randomly assign category density in these cases.

## 3 Results

We fit two linear mixed‐effects regression models to participant‐by‐trial log gaze data—one for a standard analysis window from 500 to 2000 ms post label onset and one for a data‐driven analysis window from 1000 to 2500 ms post label onset. Both models included the fixed effects of category density, perceptual richness, and vocabulary size. The models also included interactions of category density with perceptual richness, category density with vocabulary size, and perceptual richness with vocabulary size. Finally, we included maternal education as a covariate in each model. Each model also included participant and item random intercepts as well as category density between‐participant random slopes. In the remainder of this section, we directly compare the two models and interpret/follow‐up on results from the model which best fits the data.

### 3.1 Comparison of Analysis Windows

We examined several measures of model fit to determine which model to use (see Table 4). Across metrics, the standard analysis window model was a better account of variance in the dataset. The data‐driven analysis window model results are summarized in Table 5. The standard analysis window model results are summarized in Table 6. From here, we will refer to and interpret findings from the standard analysis window model.

**TABLE 4:** Comparison of model fits.

Model	*R* ^2^	AIC	BIC	Log likelihood
Data‐driven Analysis Window *1000–2500 ms post label onset*	0.06	8174.7	8246.7	−4074.4
Standard Analysis Window *500–2000 ms post label onset*	0.07	8159.8	8231.7	−4066.9

**TABLE 5:** Linear mixed effects regression results in our data‐driven analysis window from 1000 to 2500 ms after label onset.

Effect	Estimate	95% C.I.	*t*	*p*
**Intercept**	**0.36**	**0.18, 0.55**	**3.88**	**0.005** ^**^
**Category density**	**0.30**	**0.09, 0.51**	**2.79**	**0.006** ^**^
Perceptual richness	0.15	−0.15, 0.45	0.98	0.35
Vocabulary size	−0.04	−0.16, 0.07	−0.76	0.44
**Maternal education**	−**0.10**	−**0.21, 0.01**	−**1.72**	**0.09** ^*^
Category Density × Perceptual Richness	0.10	−0.28, 0.48	0.49	0.62
Category Density × Vocabulary Size	−0.04	−0.25, 0.16	−0.41	0.68
Perceptual Richness × Vocabulary Size	−0.08	−0.27, 0.11	−0.87	0.38

*Notes*: Vocabulary size is the raw count of words produced on the MBCDI. Continuous predictors (vocabulary size and maternal education) were centered and scaled. Categorical predictors were effects coded (−0.5, 0.5).

* *p* < 0.10,

** *p* < 0.01,

*** *p* < 0.001.

**TABLE 6:** Linear mixed effects regression results in our standard analysis window from 500 to 2000 ms after label onset.

Effect	Estimate	95% C.I.	*t*	*p*
**Intercept**	**0.30**	**0.08, 0.52**	**2.69**	**0.03** ^*^
**Category density**	**0.38**	**0.18, 0.59**	**3.63**	**<0.001** ^***^
Perceptual richness	0.16	−0.20, 0.49	0.81	0.43
Vocabulary size	0.00	−0.11, 0.12	0.06	0.95
Maternal education	0.04	−0.08, 0.15	0.64	0.52
Category Density × Perceptual Richness	0.04	−0.34, 0.43	0.23	0.82
Category Density × Vocabulary Size	−0.08	−0.29, 0.12	−0.80	0.43
**Perceptual Richness × Vocabulary Size**	−**0.23**	−**0.42,** −**0.04**	−**2.38**	**0.02** ^*^

*Notes*: Vocabulary size is the raw count of words produced on the MBCDI. Vocabulary size was centered and scaled. Categorical predictors were effects coded (−0.5, 0.5).

* *p* < 0.05,

*** *p* < 0.001.

### 3.2 Effects of Category Density and Perceptual Richness on Novel Word Learning

**FIGURE 4:**
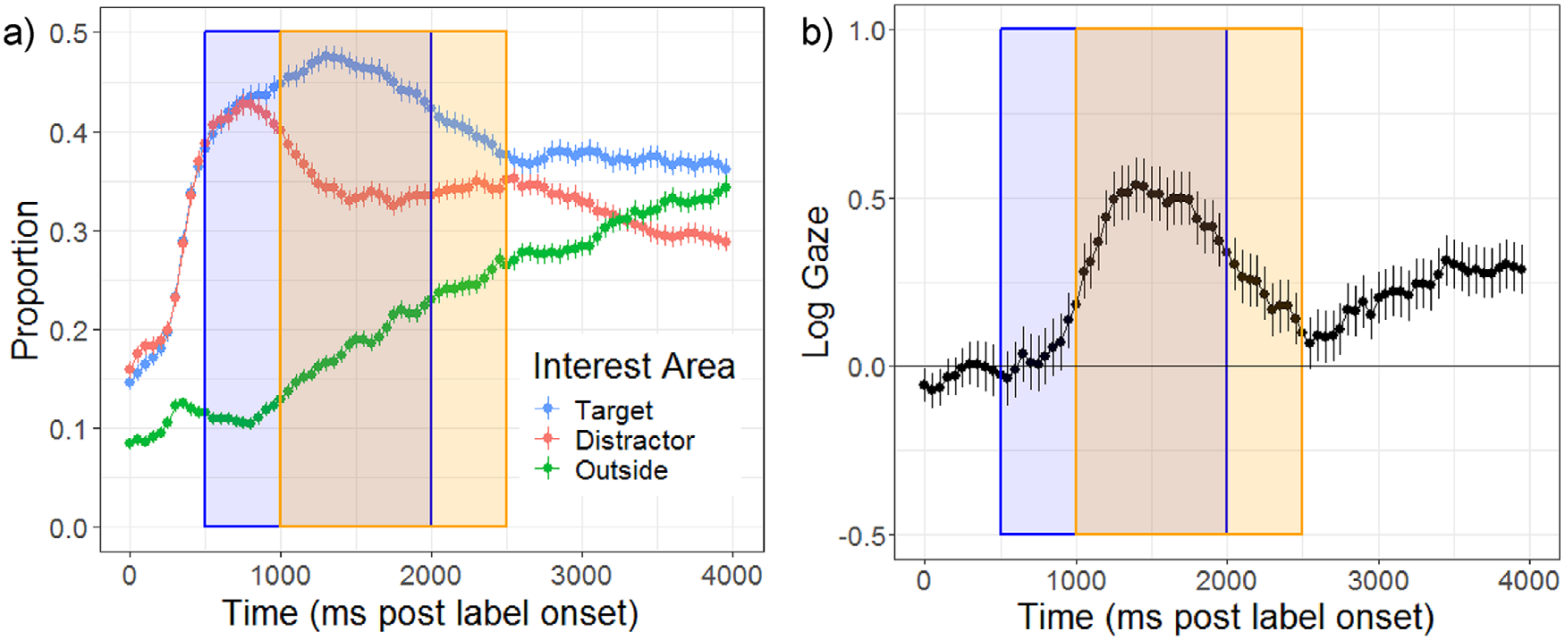
Time course of visual fixations to interest areas (a) and log gaze or the target referent relative to the distractor referent (b). The blue shaded region marks the standard analysis window. The orange shaded region marks the data‐driven analysis window. Error bars are standard error.

We expected toddlers to succeed in learning the novel word‐referent mappings, evidenced by increased looks to targets following label onset in test trials. Figure 4a depicts the proportion of looks to the IAs across the trial period. Figure 4b depicts log‐adjusted looks to the target referent relative to the distractor referent (i.e., log gaze) across the trial period. Figure 4 shows that looks to targets quickly diverge from looks to distractors, starting around 750 ms after the label onset. The advantage for targets over distractors is quantified by the model intercept (Table 6), which represents the average log gaze in the standard analysis window across conditions. This shows that overall, children looked reliably more to targets relative to distractors (Estimated Marginal Mean on log gaze scale = 0.30, *SE* = 0.11, 95% C.I. = [0.04, 0.56]). Thus, children learned and retained the novel object‐label mappings during this experiment.

**FIGURE 5:**
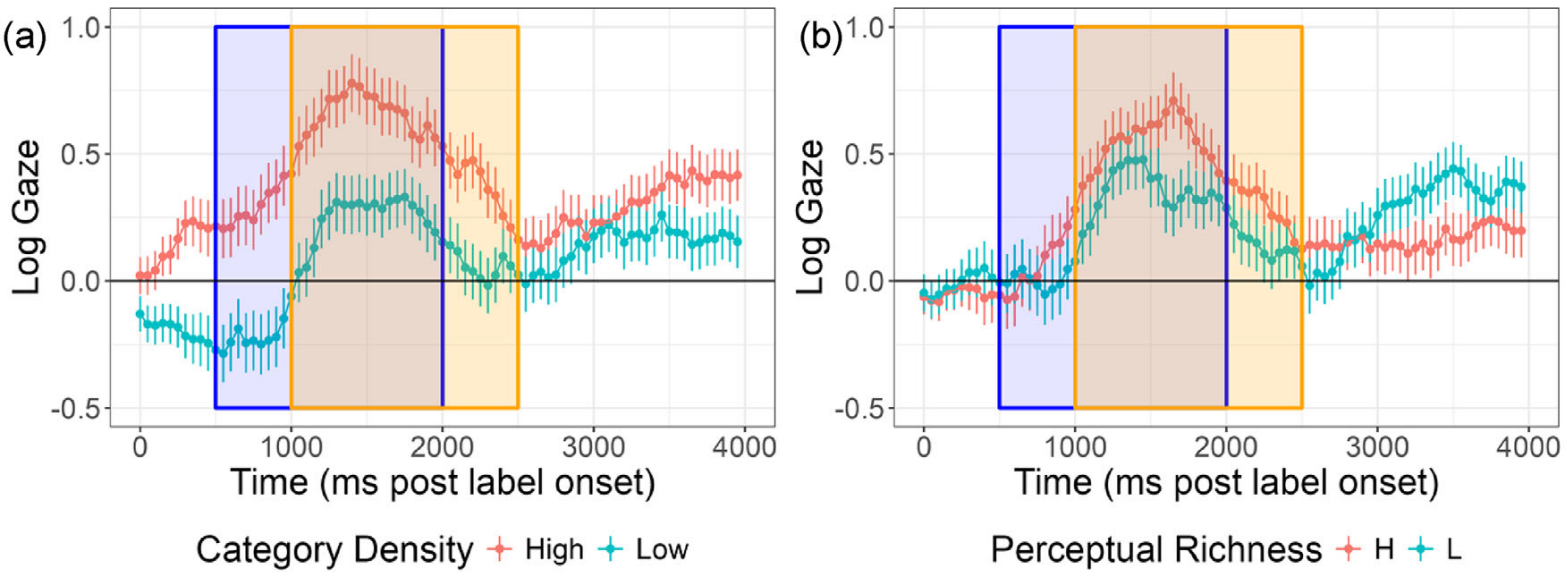
Word learning by (a) category density and (b) perceptual richness. The blue shaded region marks the standard analysis window. The orange shaded region marks the data‐driven analysis window. Error bars represent the standard error.

There were important differences in the magnitude of the target preference as a result of children's individual domain knowledge. We predicted that word learning would be facilitated by taxonomic information such that novel words from higher‐density categories would be recognized more robustly than those from lower‐density categories. This prediction was supported by the data; we observed a significant main effect of category density (see Table 6). Toddlers looked more at targets from relatively higher‐density compared to lower‐density taxonomic categories (see Table 7 for estimated marginal means). This main effect is illustrated in Figure 5a, which shows the time course of log gaze across the trial period split by levels of category density (higher or lower).

**TABLE 7:** Estimated marginal means of log gaze by category density.

	EMM	*t*	95% C.I.	*p*
Higher	0.49	4.14	[0.22, 0.76]	0.003^**^
Lower	0.11	0.87	[−0.17, 0.39]	0.40

Abbreviations: C.I., confidence interval; EMM, estimated marginal mean on log gaze scale.

** *p* < 0.01.

Finally, we expected novel words with higher counts of perceptual features to be recalled more accurately than novel words with lower counts of perceptual features, and therefore predicted a main effect of perceptual richness. We additionally speculated that the advantage of higher versus lower perceptual richness would be attenuated in higher‐density categories and therefore predicted an interaction of category density and perceptual richness. These predictions were not supported by the data; there was no significant main effect of perceptual richness or interaction of perceptual richness and category density after controlling on other model variables (see Table 6 and Figure 5b).

### 3.3 Effects of Vocabulary Size on Novel Word Learning

**FIGURE 6:**
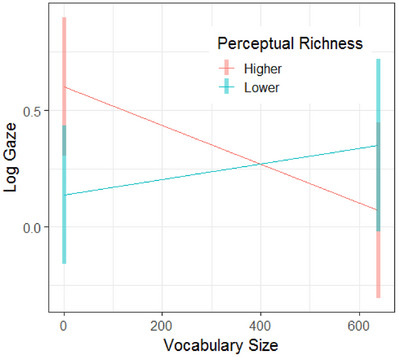
Estimated marginal means of log gaze at higher and lower levels of perceptual richness by vocabulary size.

As outlined above, we expected to find interactions between vocabulary size and category density/perceptual richness. We failed to observe an interaction of category density and vocabulary size. However, we observed a significant interaction of perceptual richness and vocabulary size (Table 6 and Figure 6), indicating that the effect of perceptual richness on novel word learning depended on toddlers’ vocabulary size. Post‐hoc comparison (Table 8) suggests that toddlers at the low‐end of the vocabulary spectrum looked more to targets relative to distractors in higher‐richness compared to lower‐richness categories. This is depicted in Figure 6; higher‐richness (red) diverges most from lower‐richness (blue) when vocabulary sizes are close to 0. When vocabulary sizes are close to 600, log gaze for higher and lower perceptual richness is closer together. Additionally, at the highest end of the vocabulary spectrum, the confidence intervals for higher and lower richness overlap. This indicates that toddlers at the high end of the vocabulary spectrum did not show a reliable effect of perceptual richness.

**TABLE 8:** Estimated slope of log gaze with respect to vocabulary size across levels of perceptual richness.

	*b*	*t*	95% C.I.	*p*
Higher–Lower	−0.14	−2.38	[−0.26, −0.02]	0.02^*^

Abbreviations: C.I., confidence interval; EMM, estimated marginal mean on log gaze scale.

* *p* < 0.05, estimates represent change in log gaze for every 100 words.

## 4 Discussion

Accounts of early word learning posit differing roles for perceptual and taxonomic dimensions of meaning. One class of accounts (see e.g., Quinn and Eimas 1997) suggests that higher‐order taxonomic structure is bootstrapped from first‐order perceptual information, while another proposes that taxonomic structure is available from the start of development (see e.g., Smiley and Brown 1979). Prior findings suggest a facilitatory effect of semantic category knowledge on word learning (Borovsky 2020; Borovsky et al. 2016a). Yet, because effects of taxonomic density had only been examined in isolation, this work made it difficult to disentangle whether these effects stem from perceptual overlap among category members or whether the effects are attributable to reasoning over higher‐order conceptual structures. To address this gap, we examined the joint contribution of taxonomic and perceptual cues to meaning on word learning at 24 months old. Three key findings emerged from this work: First, 24‐month‐olds who had a wide range of vocabulary skill successfully learned novel words. Second, toddlers leveraged their taxonomic knowledge to support word learning. That is, children more accurately identified recently taught novel objects from denser semantic categories based on their existing vocabulary knowledge. Third, children with smaller productive vocabularies used the availability of perceptual cues to support word learning, while children with larger productive vocabularies did not. We discuss the implications of each finding below.

### 4.1 Finding 1: Toddlers With a Wide Range of Language Skills Learned New Words

Across conditions, we found that toddlers in the current sample successfully learned novel words in our experiment, looking proportionally more to the target image than the distractor image after hearing the novel label. This finding is noteworthy given the wide range of vocabulary ability among our sample, which included a significant proportion of children who are classified as late talkers (LTs). In the literature on LTs (i.e., children at the low end of productive vocabulary development), studies show mixed results regarding LTs’ abilities to learn new words in similar experimental contexts. For example, Ellis Weismer et al. (2013) found that children with LT (vs. with typical development [TD]) were significantly less accurate at recalling a recently encoded word‐referent mapping to select a requested object. Similarly, in an extended word learning experiment, Macroy‐Higgins and Montemarano (2016) attempted to teach LT and TD children 12 novel words and found that LT children learned fewer words and attended less to the novel objects across experimental sessions than TD children. However, other experiments with simpler parameters suggest some equivalences in how children with LT and TD approach word learning. For instance, Ellis et al. (2015) taught 15‐month‐old children two novel word‐referent pairs in an ostensive labeling phase, where each object was labeled 14 times, and recall was tested immediately following training. Here, there were no group differences in learning accuracy. Similarly, LaTourrette et al. (2023) compared TD and LT children's ability to infer the referent of a novel noun in a sentence based on the semantic constraints of the verb (e.g., *“You can eat the dax.”*). Both groups disambiguated the novel word's referent and retained this word‐referent mapping. Taken together, these results suggest that children with smaller productive vocabularies *can* learn novel word‐referent mappings with similar accuracy to their TD peers, at least in some contexts. One key feature of our experiment and of others with positive results, is that the test phase immediately followed the learning phase. Thus, these findings point to the possibility that children at the lower end of the vocabulary learning spectrum may have difficulty retaining these mappings over time. However, further investigation is needed to verify this, as well as to understand the implications for vocabulary growth in LTs during the first few years of life.

### 4.2 Finding 2: Toddlers Leverage Taxonomic Organization to Support Word Learning

We explored the role that semantic category knowledge played in word learning while controlling for the availability of perceptual cues to meaning. From the prior work, it was unclear whether semantic density's impact on word learning could be attributed to higher‐order conceptual organization or reflected lower‐level perceptual overlap among category members. We found that, when controlling for the availability of perceptual cues to meaning, toddlers showed novel word learning advantages when they knew many other words in the semantic category to which the novel word belonged. Importantly, higher‐ and lower‐density was defined in relation to each individual child's knowledge within the semantic categories we probed. This suggests that children build on higher‐order conceptual structures to support word learning from early in development. In contrast to our a priori predictions, toddlers’ reliance on taxonomic information did not increase with larger vocabularies. This demonstrates that 24‐month‐old children, across a range of vocabulary skills, can use taxonomic structure to support word learning. More generally, these findings support the notion that taxonomic structure is available to children from early in life (see also Arias‐Trejo and Plunkett 2013; Willits et al. 2013) and, thus, provides support for semantic network building that reflects adult‐like conceptual hierarchies of knowledge.

#### 4.2.1 What Does Taxonomic Representation Buy the Learner?

Taxonomic organization of conceptual knowledge permits inductive inference (Davidson and Gelman 1990; Gelman and Markman 1986; Graham et al. 2004; Heyman and Gelman 2000; Keates and Graham 2008; Welder and Graham 2001). Identifying a new word/concept as a member within a taxonomic category allows the learner to move beyond directly observable properties to generate inferences about unobservable properties of that entity. For example, when a learner identifies a new animal as a bird, they can infer that it has certain characteristics associated with birds—such as that it lays eggs and can fly—even if they have never encountered that species of bird before. The ability to form generalizations based on category membership allows learners to quickly integrate new words/concepts into their existing conceptual structures and grow their vocabulary/conceptual knowledge. This process likely also enhances lexical and sentence‐level processing by enabling learners to retrieve their knowledge efficiently to support ongoing and incremental interpretation. This idea is consistent with findings that category density enhances language processing at the lexical and sentential level (Borovsky et al. 2016b), as well as a trove of evidence that vocabulary skill supports processing in children (Mani and Huettig 2012; Marchman et al. 2010; Marchman and Fernald 2008; Peter et al. 2019). Thus, taxonomic structures support the dynamic application of existing knowledge during language processing and learning.

#### 4.2.2 How Does Taxonomic Structure Emerge in the Lexicon?

One explanation is that children track distributional properties of the language input, which provide cues to meaning. That is, attending to the other words that co‐occur with a word can support learning about semantic structures. Returning to our introductory example, a child might infer that *lychee* and *strawberry* have similar meanings by noticing that both words are used in sentences with *juicy, eat*, and *kitchen* at similar rates. Noticing when two words co‐occur in the same linguistic contexts may induce categorization. Empirical work exploring adult artificial language learning provides support for the intuitive notion that taxonomic structures can be acquired from word co‐occurrence statistics. For example, Reeder et al. (2013) found adults successfully learned a grammatical category (X) in an artificial language with five word categories: (Q)AXB(R). In this language, A and B words provided distributional cues to the target category X, while Q and R were optional words which de‐confounded word order and co‐occurrence. Ouyang et al. (2017) found that adults inferred shared semantic content for words which co‐occurred together. Specifically, after hearing semi‐artificial sentences where novel words appeared with words from a single semantic category (providing a coherent semantic context), adults selected category‐congruent images as referents for the novel items. These studies show adults track distributional statistics to learn syntactic and semantic categories.

A host of developmental evidence similarly supports this distributional learning account of taxonomic structures. From infancy, children track probabilistic distributions across their language input (see Saffran and Kirkham 2018 for a review). Young children also track distributional statistics across learning instances (e.g., word‐object co‐occurrences; Smith and Yu 2008). Further support comes from approaches that look for distributional cues to word meaning in child‐directed language input (Huebner and Willits 2018; Unger et al. 2023). For instance, Huebner and Willits (2018) trained two simple recurrent neural networks to perform next word prediction on a corpus of child‐directed speech (CDS). In addition to the models performing well on the next‐word prediction task, an examination of the models’ hidden layers revealed principal components that encoded semantic information (e.g., activity context). Unger et al. (2023) took a simpler approach and explored the frequency of word pair co‐occurrences in CDS. The authors found that words from the same semantic category reliably co‐occurred together in CDS. Recent experimental work has found that both direct word co‐occurrences (e.g., eat‐apple) and shared contexts (e.g., both apple and pear appear in sentences with eat) support word learning in adults (Savic et al. 2022). That is, exposure to language input containing these regularities supported the acquisition of novel words, evidenced by the finding that novel words learned in this context primed semantically related familiar words. Thus, it is possible that children may be able to uncover at least some of the higher‐order structure of concepts by tracking distributional properties of the language input.

The literature summarized in the paragraph above concerns the ability to track a single word‐label mapping over time. Our summary of this work assumes that the higher‐order structures need not be known for this distributional learning process to proceed. However, once the taxonomic categories are known, this information may in turn support cross‐situational word learning, representing a bidirectional relationship. That is, our findings would suggest that children consider what they know about a semantic category when they encounter new words to help uncover elements of word meaning. This information could support word learning within the kind of everyday (ambiguous) learning environments that the cross‐situational word learning literature seeks to explain. This hypothesis concretely posits that children track distributions at multiple levels—a simple word‐referent level and a word‐category level. What's more, it is possible that word‐label mappings embedded in more semantically rich contexts (either richness created by the environment or by the child's existing knowledge) may be subject to less decay over time. Thus, we are suggesting here that taxonomic structure (once acquired) may result in more accurate initial word‐label mappings during cross‐situational learning that are more robustly retained over time. We note, however, that this is only speculative and presents potential for future investigation.

Yet, children are not simply passive recipients of environmental input, but actively guide their own learning too. Curiosity accounts of conceptual development have found that children learn a new property of an object more readily when they are interested in that individual object (Begus et al. 2014; Begus and Southgate 2012) and learn novel words more robustly when they are more interested in the semantic category that the novel word belongs to (Ackermann et al. 2020). What's more, children enrolled in play‐based educational programs (e.g., Montessori schools) where learning is guided by child interest, develop deeper and more interconnected semantic representations (Denervaud et al. 2021). This suggests that child interest may play a critical role in the acquisition of adult‐like conceptual knowledge.

### 4.3 Finding 3: Perceptual Richness and Vocabulary Size Interact to Predict Word Learning

Previous work modeling the normative growth of early acquired semantic knowledge indicates that words/concepts with a greater number of perceptual features are acquired earlier in development than words/concepts with fewer available perceptual features (Kueser and Borovsky 2024; Peters and Borovsky 2019). It was on this basis that we predicted that the availability of more perceptual cues to meaning would better support word learning. While the data did not support a universal perceptual richness “boost” for word learning across children, we found that perceptual richness differentially supported toddlers’ learning as a function of vocabulary size. Specifically, we found that children with smaller productive vocabularies profited from the availability of perceptual cues, but children with larger productive vocabularies did not.

We outlined two potential developmental trends regarding the role of perceptual features in word learning in our Introduction. On one account, the *perceptual‐to‐conceptual* shift (Quinn and Eimas 1997), children's early conceptual representations are organized by perceptual features. As children acquire more concepts, they undergo a process of conceptual reorganization to represent concepts in terms of higher‐order structures. As discussed above, the strong claim this account makes is unsupported by the current evidence. However, a weaker version of this claim seems plausible given the nuance observed here. That is, it appears that the availability of perceptual features supports word learning at the start of development. Indeed, researchers agree that having referents which are concrete and imageable makes word learning easier (Caselli and Pyers 2017b; Della Rosa et al. 2010b; Gilhooly and Logie 1980b; Hansen 2017b; McDonough et al. 2011; Perry et al. 2018; Pruden et al. 2006).

Yet another possibility, also consistent with our data, is that using taxonomic information to support word learning involves the generation of inferences about meanings. Perhaps children have access to abstract, higher‐order conceptual structures from the start of development, but need to learn to generate the appropriate inferences. In contrast, perceptual information is directly accessible and does not require inference generation, which may explain why it is more readily used to support learning at earlier stages in development. Over time, children may come to rely more on abstract meanings than surface‐level properties, given the predictive value of these more abstract features in reasoning about events in the world. In other words, perceptual information may help children initially integrate new semantic knowledge, but is unlikely to help children reason more broadly about the world, and so children need to learn to use other dimensions of meaning too. For example, knowing that a bird has feathers does not itself indicate that it will fly. Importantly, this account does not require conceptual restructuring; rather, it highlights that how information is used in learning may reflect shifts in sensitivities as opposed to changes in the underlying conceptual architecture.

These findings pattern with recent work about toddlers’ ability to process language under conditions of semantic interference (Kueser et al., under review). Kueser and colleagues conducted an eye‐tracked lexical recognition task with 18‐month‐old children. The authors manipulated whether target and distractor object pairs were semantically unrelated (U: DOG‐APPLE), perceptually related (PR: DOG‐TABLE, both have four legs), or taxonomically and perceptually related (TPR: DOG‐HORSE, both are animals and have four legs). They found TD infants experienced interference from taxonomic information, recognizing words less accurately in the TPR trials, but did not experience interference from perceptual information alone. In contrast, LT infants experienced semantic interference during both PR and TPR trials. This finding, as well as our results, suggest that children with smaller productive vocabularies attend more to perceptual aspects of meaning than children with larger productive vocabularies.

These findings also highlight an important developmental question: Do the observed differences in how children with less advanced vocabulary development attend to perceptual dimensions of meaning reflect a typical developmental trajectory, or are they indicative of an underlying language‐learning difficulty? This pattern can be viewed as a delay in language learning: variation in early word learning abilities may reflect individual differences in the pace of learning, where LT children have less access to taxonomic information simply because they know fewer words. This pattern could also suggest a deficit in children at the low end of vocabulary growth: they may fail to attend to the most informative dimensions of meaning (i.e., taxonomic categories), a bias that may slow further growth in vocabulary and potentially point to risk factors for developing language difficulties during the school years. This is potentially aligned with studies which find that LT and TD children differ in their use of a shape bias to guide word learning. Specifically, TD children typically extend labels to novel exemplars based on shape, whereas LT children fail to extend labels systematically on the basis of object shape (Colunga and Sims 2017; Jones 2003; Perry and Kucker 2019). However, keeping in mind recent work demonstrating that perceptual features predict the normative age of acquisition for many early‐acquired words (Peters and Borovsky 2019), we argue that these findings more likely reflect the continuous nature of language learning.

To disentangle the question posed above, future work is needed to better understand the role that perceptual information plays in early word learning and conceptual development. One might ask whether children with smaller productive vocabularies use perceptual cues to meaning as additional supports for language learning or whether a lack of perceptual information would inhibit their ability to learn new words. One potential approach to answer this question is to explore the role of perceptual cues to meaning in word learning across development, for example, throughout the second year of life. There is significant insight to be gained from evaluating whether perceptual information provides an advantage in word learning at earlier stages of development. Either cross‐sectional or longitudinal approaches which examine word learning across a period of 12 to 24 months old can help unravel these questions of deficit or delay. On the delay view, we would predict that the effect of perceptual richness on word learning would decrease in LTs as they learn more words. On the deficits view, we would predict a consistent effect of perceptual information on word learning in LTs across a wide age range. This would provide insight as to whether perceptual elements of meaning serve as more reliable cues for word learning at different stages of development.

### 4.4 Limitations

We note several limitations to the current design. First, we tested novel word‐referent mappings following an ostensive learning phase, wherein novel words were presented as isolated utterances. This approach—coined “the map trap” by Wojcik et al. (2022)—fails to capture the complexity of word learning in naturalistic contexts, where children encounter new words in the presence of complex visual referential environments and embedded in rich linguistic input (Yu et al. 2021). Second, we did not assess retention of the object‐label mapping over time. As noted above, the ability to retain word‐referent associations across time may vary with language skill. Finally, while we attempted to remove novel items the toddlers had prior experience with, this relied on parental report. Therefore, it is possible that children had some prior experience with the novel objects and/or labels used in this experiment.

## 5 Conclusions

The current experiment explored the joint contribution of taxonomic and perceptual dimensions of meaning on word learning in a group of 24‐month‐olds. We found that toddlers across a range of vocabulary skills learned novel words. We also found that word learning was facilitated when novel items belonged to semantic categories which children had more knowledge in. Finally, we found that perceptual information provided an additional boost to word learning for children with smaller vocabularies. Together, these findings suggest that structured taxonomic knowledge is a better predictor of word learning compared to lower‐level perceptual features in 24‐month‐old learners. However, perceptual cues to meaning may provide additional support for vocabulary growth at the start of development, particularly for learners with smaller vocabularies.

## Author Contributions

**Amanda Rose Yuile**: data curation, formal analysis, investigation, validation, visualization, writing – original draft, writing – review and editing. **Justin B. Kueser**: conceptualization, data curation, investigation, methodology, software, validation, writing – review and editing. **Claney Outzen**: conceptualization, data curation, investigation, methodology, project administration, resources, software, validation. **Sharon Christ**: contributed to formal analysis, funding acquisition, and validation. **Risa Stiegler**: contributed to data curation, investigation, methodology, project administration. **MaryCarson Adams**: contributed to data curation, investigation, methodology, project administration. **Barbara Brown**: contributed to investigation. **Arielle Borovsky**: contributed to conceptualization, funding acquisition, methodology, project administration, resources, software, supervision, writing – review and editing.

## Conflicts of Interest

The authors declare no conflicts of interest.

## Funding

All authors were supported by R01 DC018593.

## Data Availability

The data and analysis scripts are available at https://osf.io/5jwcu/.

## Endnotes

1 The current work is unable to speak with specificity as to the nature of these underlying conceptual representations (e.g., discrete categories vs. correlations of shared features), and so we choose to remain agnostic about this concern in our writing here.

2 Eleven participants had visits that were more than 2 weeks apart because toddlers got sick and/or families had scheduling constraints.

3 One participant was accidentally recorded with a sampling rate of 1000 Hz. The data from this participant were downsampled just as the other participants’ data following the procedure in Data Processing.

4 Due to an experimenter misunderstanding, a subsample of participants was collected using a “drift correction” procedure in which a linear correction was applied to the calibration model based on a single, central point of fixation.

5 At the suggestion of a reviewer, we also conducted a cluster‐based permutation test comparing log gaze to chance level (0) in each 50 ms time bin from label onset to 4000 ms after label onset. In this analysis, we observed two significant clusters: 1050–2400 ms (cumulative *F* = 537.72, *p* < 0.001) and 3050–3950 (cumulative *F* = 174.07, *p* < 0.001). The first cluster has the largest cumulative statistic and closely aligns with the window identified using our rolling average approach.

## References

[desc70087-bib-0001] Ackermann, L. , R. Hepach , and N. Mani . 2020. “Children Learn Words Easier When They Are Interested in the Category to Which the Word Belongs.” Developmental Science 23, no. 3: e12915. 10.1111/desc.12915. 31618505

[desc70087-bib-0002] Arias‐Trejo, N. , and K. Plunkett . 2009. “Lexical–Semantic Priming Effects During Infancy.” Philosophical Transactions of the Royal Society B: Biological Sciences 364, no. 1536: 3633–3647. 10.1098/rstb.2009.0146. PMC2846315 19933137

[desc70087-bib-0003] Arias‐Trejo, N. , and K. Plunkett . 2013. “What's in a Link: Associative and Taxonomic Priming Effects in the Infant Lexicon.” Cognition 128, no. 2: 214–227. 10.1016/j.cognition.2013.03.008. 23688648

[desc70087-bib-0004] Bates, D. , M. Mächler , B. Bolker , and S. Walker . 2015. “Fitting Linear Mixed‐Effects Models Using lme4.” Journal of Statistical Software 67, no. 1: 1–48. 10.18637/jss.v067.i01.

[desc70087-bib-0005] Bayley, N. , and G. P. Aylward . 2019. Bayley Scales of Infant and Toddler Development. 4th ed. Pearson Education.

[desc70087-bib-0006] Begus, K. , T. Gliga , and V. Southgate . 2014. “Infants Learn What They Want to Learn: Responding to Infant Pointing Leads to Superior Learning.” PLoS ONE 9, no. 10: e108817. 10.1371/journal.pone.0108817. 25290444 PMC4188542

[desc70087-bib-0007] Begus, K. , and V. Southgate . 2012. “Infant Pointing Serves an Interrogative Function.” Developmental Science 15, no. 5: 611–617. 10.1111/j.1467-7687.2012.01160.x. 22925509

[desc70087-bib-0008] Bion, R. A. H. , A. Borovsky , and A. Fernald . 2013. “Fast Mapping, Slow Learning: Disambiguation of Novel Word–Object Mappings in Relation to Vocabulary Learning at 18, 24, and 30 months.” Cognition 126, no. 1: 39–53. 10.1016/j.cognition.2012.08.008. 23063233 PMC6590692

[desc70087-bib-0009] Borovsky, A. 2020. “When Slowing Down Processing Helps Learning: Lexico‐Semantic Structure Supports Retention, but Interferes With Disambiguation of Novel Object‐Label Mappings.” Developmental Science 23, no. 6: e12963. 10.1111/desc.12963. 32160363 PMC8128105

[desc70087-bib-0010] Borovsky, A. , E. M. Ellis , J. L. Evans , and J. L. Elman . 2016a. “Lexical Leverage: Category Knowledge Boosts Real‐Time Novel Word Recognition in 2‐Year‐Olds.” Developmental Science 19, no. 6: 918–932. 10.1111/desc.12343. 26452444 PMC4826629

[desc70087-bib-0011] Borovsky, A. , E. M. Ellis , J. L. Evans , and J. L. Elman . 2016b. “Semantic Structure in Vocabulary Knowledge Interacts With Lexical and Sentence Processing in Infancy.” Child Development 87, no. 6: 1893–1908. 10.1111/cdev.12554. 27302575 PMC5159320

[desc70087-bib-0012] Borovsky, A. , R. E. Peters , J. I. Cox , and K. McRae . 2023. “Feats: A Database of Semantic Features for Early Produced Noun Concepts.” Behavior Research Methods 56, no. 4: 3259–3279. 10.3758/s13428-023-02242-x. 38148439 PMC11630474

[desc70087-bib-0013] Caselli, N. K. , and J. E. Pyers . 2017a. “The Road to Language Learning Is Not Entirely Iconic: Iconicity, Neighborhood Density, and Frequency Facilitate Acquisition of Sign Language.” Psychological Science 28, no. 7: 979–987. 10.1177/0956797617700498. 28557672 PMC5507709

[desc70087-bib-0014] Caselli, N. K. , and J. E. Pyers . 2017b. “The Road to Language Learning Is Not Entirely Iconic: Iconicity, Neighborhood Density, and Frequency Facilitate Acquisition of Sign Language.” Psychological Science 28, no. 7: 979–987. 10.1177/0956797617700498. 28557672 PMC5507709

[desc70087-bib-0015] Chow, J. , A. M. Aimola Davies , L. J. Fuentes , and K. Plunkett . 2019. “The Vocabulary Spurt Predicts the Emergence of Backward Semantic Inhibition in 18‐Month‐Old Toddlers.” Developmental Science 22, no. 2: e12754. 10.1111/desc.12754. 30248216

[desc70087-bib-0016] Coffey, J. R. , M. Zeitlin , J. Crawford , and J. Snedeker . 2024. “It's All in the Interaction: Early Acquired Words Are both Frequent and Highly Imageable.” Open Mind 8: 309–332. 10.1162/opmi_a_00130. 38571529 PMC10990573

[desc70087-bib-0017] Colunga, E. , and C. E. Sims . 2017. “Not Only Size Matters: Early‐Talker and Late‐Talker Vocabularies Support Different Word‐Learning Biases in Babies and Networks.” Cognitive Science 41, no. S1: 73–95. 10.1111/cogs.12409. 27873349 PMC6039116

[desc70087-bib-0018] Colunga, E. , and L. B. Smith . 2005. “From the Lexicon to Expectations about Kinds: A Role for Associative Learning.” Psychological Review 112, no. 2: 347–382. 10.1037/0033-295X.112.2.347. 15783290

[desc70087-bib-0019] Cree, G. S. , C. McNorgan , and K. McRae . 2006. “Distinctive Features Hold a Privileged Status in the Computation of Word Meaning: Implications for Theories of Semantic Memory.” Journal of Experimental Psychology: Learning, Memory, and Cognition 32, no. 4: 643–658. 10.1037/0278-7393.32.4.643. 16822138 PMC3226832

[desc70087-bib-0020] Cree, G. S. , and K. McRae . 2003. “Analyzing the Factors Underlying the Structure and Computation of the Meaning of Chipmunk, Cherry, Chisel, Cheese, and Cello (and many other such concrete nouns).” Journal of Experimental Psychology: General 132, no. 2: 163–201. 10.1037/0096-3445.132.2.163. 12825636

[desc70087-bib-0021] Davidson, N. S. , and S. A. Gelman . 1990. “Inductions From Novel Categories: The Role of Language and Conceptual Structure.” Cognitive Development 5, no. 2: 151–176. 10.1016/0885-2014(90)90024-N.

[desc70087-bib-0022] Rosa, P. A. D. , E. Catricalà , G. Vigliocco , and S. F. Cappa . 2010a. “Beyond the Abstract—Concrete Dichotomy: Mode of Acquisition, Concreteness, Imageability, Familiarity, Age of Acquisition, Context Availability, and Abstractness Norms for a Set of 417 Italian Words.” Behavior Research Methods 42, no. 4: 1042–1048. 10.3758/BRM.42.4.1042. 21139171

[desc70087-bib-0023] Rosa, P. A. D. , E. Catricalà , G. Vigliocco , and S. F. Cappa . 2010b. “Beyond the Abstract—Concrete Dichotomy: Mode of Acquisition, Concreteness, Imageability, Familiarity, Age of Acquisition, Context Availability, and Abstractness Norms for a Set of 417 Italian Words.” Behavior Research Methods 42, no. 4: 1042–1048. 10.3758/BRM.42.4.1042. 21139171

[desc70087-bib-0024] Denervaud, S. , A. P. Christensen , Y. N. Kenett , and R. E. Beaty . 2021. “Education Shapes the Structure of Semantic Memory and Impacts Creative Thinking.” NPJ Science of Learning 6, no. 1: 35. 10.1038/s41539-021-00113-8. 34887430 PMC8660875

[desc70087-bib-0025] Ellis, E. M. , A. Borovsky , J. L. Elman , and J. L. Evans . 2015. “Novel Word Learning: An Eye‐Tracking Study. Are 18‐Month‐Old Late Talkers Really Different From Their Typical Peers?” Journal of Communication Disorders 58: 143–157. 10.1016/j.jcomdis.2015.06.011. 26188415 PMC4659719

[desc70087-bib-0026] Ellis Weismer, S. , C. E. Venker , J. L. Evans , and M. J. Moyle . 2013. “Fast Mapping in Late‐Talking Toddlers.” Applied Psycholinguistics 34, no. 1: 69–89. 10.1017/S0142716411000610. 23898218 PMC3724169

[desc70087-bib-0085] Fenson, L. , V. A. Marchman , D. J. Thal , P. S. Dale , J. S. Reznik , and E. Bates . 2007. MacArthur‐Bates Communicative Development Inventories: User’s Guide and Technical Manual. 2nd ed. Brookes Publishing Co.

[desc70087-bib-0027] Fernald, A. , R. Zangl , A. Portillo , and V. Marchman . 2008. “Looking While Listening: Using Eye Movements to Monitor Spoken Language Comprehension by Infants and Young Children.” In Developmental Psycholinguistics: On‐line methods in children's language processing edited by I. Sekerina , E. Fernández , and H. Clahsen . 97–135. John Benjamins Publishing Company. 10.1075/lald.44.06fer.

[desc70087-bib-0028] Gelman, S. A. , and E. M. Markman . 1986. “Categories and Induction in Young Children.” Cognition 23, no. 3: 183–209. 10.1016/0010-0277(86)90034-X. 3791915

[desc70087-bib-0029] Gilhooly, K. J. , and R. H. Logie . 1980a. “Age‐of‐Acquisition, Imagery, Concreteness, Familiarity, and Ambiguity Measures for 1,944 Words.” Behavior Research Methods & Instrumentation 12, no. 4: 395–427. 10.3758/BF03201693.

[desc70087-bib-0030] Gilhooly, K. J. , and R. H. Logie . 1980b. “Meaning‐Dependent Ratings of Imagery, Age of Acquisition, Familiarity, and Concreteness for 387 Ambiguous Words.” Behavior Research Methods & Instrumentation 12, no. 4: 428–450. 10.3758/BF03201694.

[desc70087-bib-0031] Gleitman, L. R. , and J. C. Trueswell . 2020. “Easy Words: Reference Resolution in a Malevolent Referent World.” Topics in Cognitive Science 12, no. 1: 22–47. 10.1111/tops.12352. 29908001 PMC6925650

[desc70087-bib-0032] Gopnik, A. , S. Choi , and T. Baumberger . 1996. “Cross‐Linguistic Differences in Early Semantic and Cognitive Development.” Cognitive Development 11, no. 2: 197–225. 10.1016/S0885-2014(96)90003-9.

[desc70087-bib-0033] Gopnik, A. , and A. N. Meltzoff . 1992. “Categorization and Naming: Basic‐Level Sorting in Eighteen‐Month‐Olds and Its Relation to Language.” Child Development 63, no. 5: 1091. 10.2307/1131520.

[desc70087-bib-0034] Graham, S. A. , C. S. Kilbreath , and A. N. Welder . 2004. “Thirteen‐Month‐Olds Rely on Shared Labels and Shape Similarity for Inductive Inferences.” Child Development 75, no. 2: 409–427. 10.1111/j.1467-8624.2004.00683.x. 15056196

[desc70087-bib-0035] Grondin, R. , S. J. Lupker , and K. McRae . 2009. “Shared Features Dominate Semantic Richness Effects for Concrete Concepts.” Journal of Memory and Language 60, no. 1: 1–19. 10.1016/j.jml.2008.09.001. 20046224 PMC2634287

[desc70087-bib-0036] Hadley, E. B. , D. K. Dickinson , K. Hirsh‐Pasek , and R. M. Golinkoff . 2019. “Building Semantic Networks: The Impact of a Vocabulary Intervention on Preschoolers' Depth of Word Knowledge.” Reading Research Quarterly 54, no. 1: 41–61. 10.1002/rrq.225.

[desc70087-bib-0037] Hansen, P. 2017a. “What Makes a Word Easy to Acquire? The Effects of Word Class, Frequency, Imageability and Phonological Neighbourhood Density on Lexical Development.” First Language 37, no. 2: 205–225. 10.1177/0142723716679956.

[desc70087-bib-0038] Hansen, P. 2017b. “What Makes a Word Easy to Acquire? The Effects of Word Class, Frequency, Imageability and Phonological Neighbourhood Density on Lexical Development.” First Language 37, no. 2: 205–225. 10.1177/0142723716679956.

[desc70087-bib-0039] Heyman, G. D. , and S. A. Gelman . 2000. “Preschool Children's Use of Trait Labels to Make Inductive Inferences.” Journal of Experimental Child Psychology 77, no. 1: 1–19. 10.1006/jecp.1999.2555. 10964456

[desc70087-bib-0040] Hills, T. T. , M. Maouene , J. Maouene , A. Sheya , and L. Smith . 2009a. “Categorical Structure Among Shared Features in Networks of Early‐Learned Nouns.” Cognition 112, no. 3: 381–396. 10.1016/j.cognition.2009.06.002. 19576579 PMC2734996

[desc70087-bib-0041] Hills, T. T. , M. Maouene , J. Maouene , A. Sheya , and L. Smith . 2009b. “Longitudinal Analysis of Early Semantic Networks: Preferential Attachment or Preferential Acquisition?” Psychological Science 20, no. 6: 729–739. 10.1111/j.1467-9280.2009.02365.x. 19470123 PMC4216730

[desc70087-bib-0042] Huebner, P. A. , and J. A. Willits . 2018. “Structured Semantic Knowledge Can Emerge Automatically From Predicting Word Sequences in Child‐Directed Speech.” Frontiers in Psychology 9: 133. 10.3389/fpsyg.2018.00133. 29520243 PMC5827184

[desc70087-bib-0043] Jones, S. S. 2003. “Late Talkers Show no Shape Bias in a Novel Name Extension Task.” Developmental Science 6, no. 5: 477–483. 10.1111/1467-7687.00304.

[desc70087-bib-0044] Keates, J. , and S. A. Graham . 2008. “Category Markers or Attributes: Why Do Labels Guide Infants' Inductive Inferences?” Psychological Science 19, no. 12: 1287–1293. 10.1111/j.1467-9280.2008.02237.x. 19121139

[desc70087-bib-0045] Kueser, J. B. , and A. Borovsky . 2024. “Complex Meanings Shape Early Noun and Verb Vocabulary Structure and Learning.” Network Science [Special issue]. *Canadian Journal of Experimental Psychology* 79, no. 1: 85–97. https://osf.io/s5zda/. 10.1037/cep0000355 PMC11908919 39432347

[desc70087-bib-0046] Kueser, J. B. , S. Horvath , and A. Borovsky . 2023. “Two Pathways in Vocabulary Development: Large‐Scale Differences in Noun and Verb Semantic Structure.” Cognitive Psychology 143: 101574. 10.1016/j.cogpsych.2023.101574. 37209501 PMC10832511

[desc70087-bib-0047] Landau, B. , L. B. Smith , and S. S. Jones . 1988. “The Importance of Shape in Early Lexical Learning.” Cognitive Development 3, no. 3: 299–321. 10.1016/0885-2014(88)90014-7.

[desc70087-bib-0048] LaTourrette, A. , S. Waxman , L. S. Wakschlag , E. S. Norton , and A. Weisleder . 2023. “From Recognizing Known Words to Learning New Ones: Comparing Online Speech Processing in Typically Developing and Late‐Talking 2‐Year‐Olds.” Journal of Speech, Language, and Hearing Research 66, no. 5: 1658–1677. 10.1044/2023_JSLHR-22-00580. PMC10457094 36989138

[desc70087-bib-0049] Lenth, R. 2019. Emmeans: Estimated Marginal Means, aka Least‐Squares Means . https://CRAN.R‐project.org/package=emmeans.

[desc70087-bib-0050] Macroy‐Higgins, M. , and E. A. Montemarano . 2016. “Attention and Word Learning in Toddlers Who Are Late Talkers.” Journal of Child Language 43, no. 5: 1020–1037. 10.1017/S0305000915000379. 27464621

[desc70087-bib-0051] Mani, N. , and F. Huettig . 2012. “Prediction During Language Processing Is a Piece of Cake—But Only for Skilled Producers.” Journal of Experimental Psychology: Human Perception and Performance 38, no. 4: 843–847. 10.1037/a0029284. 22774799

[desc70087-bib-0052] Marchman, V. A. , and A. Fernald . 2008. “Speed of Word Recognition and Vocabulary Knowledge in Infancy Predict Cognitive and Language Outcomes in Later Childhood.” Developmental Science 11, no. 3. 10.1111/j.1467-7687.2008.00671.x. PMC2905590 18466367

[desc70087-bib-0053] Marchman, V. A. , A. Fernald , and N. Hurtado . 2010. “How Vocabulary Size in Two Languages Relates to Efficiency in Spoken Word Recognition by Young Spanish–English Bilinguals.” Journal of Child Language 37, no. 4: 817–840. 10.1017/S0305000909990055. 19726000 PMC2910833

[desc70087-bib-0054] McDonough, C. , L. Song , K. Hirsh‐Pasek , R. M. Golinkoff , and R. Lannon . 2011. “An Image Is Worth a Thousand Words: Why Nouns Tend to Dominate Verbs in Early Word Learning.” Developmental Science 14, no. 2: 181–189. 10.1111/j.1467-7687.2010.00968.x. 21359165 PMC3043374

[desc70087-bib-0055] McNorgan, C. , R. A. Kotack , D. C. Meehan , and K. McRae . 2007. “Feature‐Feature Causal Relations and Statistical Co‐Occurrences in Object Concepts.” Memory & Cognition 35, no. 3: 418–431. 10.3758/BF03193282. 17691142 PMC3269815

[desc70087-bib-0056] McRae, K. , G. S. Cree , M. S. Seidenberg , and C. Mcnorgan . 2005. “Semantic Feature Production Norms for a Large Set of Living and Nonliving Things.” Behavior Research Methods 37, no. 4: 547–559. 10.3758/BF03192726. 16629288

[desc70087-bib-0057] Ouyang, L. , L. Boroditsky , and M. C. Frank . 2017. “Semantic Coherence Facilitates Distributional Learning.” Cognitive Science 41, no. S4: 855–884. 10.1111/cogs.12360. 26988338

[desc70087-bib-0058] Perry, L. K. , and S. C. Kucker . 2019. “The Heterogeneity of Word Learning Biases in Late‐Talking Children.” Journal of Speech, Language, and Hearing Research 62, no. 3: 554–563. 10.1044/2019_JSLHR-L-ASTM-18-0234. 30950748

[desc70087-bib-0059] Perry, L. K. , M. Perlman , B. Winter , D. W. Massaro , and G. Lupyan . 2017. “Iconicity in the Speech of Children and Adults.” Developmental Science 21, no. 3: e12572. 10.1111/desc.12572. 28523758

[desc70087-bib-0060] Perry, L. K. , M. Perlman , B. Winter , D. W. Massaro , and G. Lupyan . 2018. “Iconicity in the Speech of Children and Adults.” Developmental Science 21, no. 3: e12572. 10.1111/desc.12572. 28523758

[desc70087-bib-0061] Peter, M. S. , S. Durrant , A. Jessop , A. Bidgood , J. M. Pine , and C. F. Rowland . 2019. “Does Speed of Processing or Vocabulary Size Predict Later Language Growth in Toddlers?” Cognitive Psychology 115: 101238. 10.1016/j.cogpsych.2019.101238. 31539813

[desc70087-bib-0062] Peters, R. , and A. Borovsky . 2019. “Modeling Early Lexico‐Semantic Network Development: Perceptual Features Matter Most.” Journal of Experimental Psychology: General 148, no. 4: 763–782. 10.1037/xge0000596. 30973265 PMC6461380

[desc70087-bib-0063] Peters, R. E. , J. B. Kueser , and A. Borovsky . 2021. “Perceptual Connectivity Influences Toddlers' Attention to Known Objects and Subsequent Label Processing.” Brain Sciences 11, no. 2: 163. 10.3390/brainsci11020163. 33513707 PMC7912090

[desc70087-bib-0064] Poulin‐Dubois, D. , S. Graham , and L. Sippola . 1995. “Early Lexical Development: The Contribution of Parental Labelling and Infants' Categorization Abilities.” Journal of Child Language 22, no. 2: 325–343. 10.1017/S0305000900009818. 8550726

[desc70087-bib-0065] Pruden, S. M. , K. Hirsh‐Pasek , R. M. Golinkoff , and E. A. Hennon . 2006. “The Birth of Words: Ten‐Month‐Olds Learn Words Through Perceptual Salience.” Child Development 77, no. 2: 266–280. 10.1111/j.1467-8624.2006.00869.x. 16611171 PMC4621011

[desc70087-bib-0066] Quinn, P. C. , and P. D. Eimas . 1997. “A Reexamination of the Perceptual‐to‐Conceptual Shift in Mental Representations.” Review of General Psychology 1, no. 3: 271–287. 10.1037/1089-2680.1.3.271.

[desc70087-bib-0067] R Development Core Team . 2008. *R: A Language and Environment for Statistical Computing*. R Foundation for Statistical Computing. http://www.R‐project.org.

[desc70087-bib-0084] Rakison, D. H. 2003. “Parts, motion, and the development of the animate‐inanimate distinction in infancy.” In Early category and concept development: Making sense of the blooming, buzzing confusion, edited by D. H. Rakison and L. M. Oakes . 159–192. Oxford University Press.

[desc70087-bib-0068] Rämä, P. , L. Sirri , and J. Serres . 2013. “Development of Lexical–Semantic Language System: N400 Priming Effect for Spoken Words in 18‐ and 24‐Month‐Old Children.” Brain and Language 125, no. 1: 1–10. 10.1016/j.bandl.2013.01.009. 23435193

[desc70087-bib-0069] Reeder, P. A. , E. L. Newport , and R. N. Aslin . 2013. “From Shared Contexts to Syntactic Categories: The Role of Distributional Information in Learning Linguistic Form‐Classes.” Cognitive Psychology 66, no. 1: 30–54. 10.1016/j.cogpsych.2012.09.001. 23089290 PMC3621024

[desc70087-bib-0070] Saffran, J. R. , and N. Z. Kirkham . 2018. “Infant Statistical Learning.” Annual Review of Psychology 69, no. 1: 181–203. 10.1146/annurev-psych-122216-011805. PMC5754249 28793812

[desc70087-bib-0071] Savic, O. , L. Unger , and V. M. Sloutsky . 2022. “Exposure to Co‐Occurrence Regularities in Language Drives Semantic Integration of New Words.” Journal of Experimental Psychology: Learning, Memory, and Cognition 48, no. 7: 1064–1081. 10.1037/xlm0001122. 35389699 PMC10243553

[desc70087-bib-0072] Schopler, E. , and M. E. Van Bourgondien . 2010. Childhood Autism Rating Scale. 2nd ed. [Computer software]. Western Psychological Services.

[desc70087-bib-0073] Seidl, A. H. , M. Indarjit , and A. Borovsky . 2024. “Touch to Learn: Multisensory Input Supports Word Learning and Processing.” Developmental Science 27, no. 1: e13419. 10.1111/desc.13419. 37291692 PMC10704002

[desc70087-bib-0074] Smiley, S. S. , and A. L. Brown . 1979. “Conceptual Preference for Thematic or Taxonomic Relations: A Nonmonotonic Age Trend From Preschool to Old Age.” Journal of Experimental Child Psychology 28, no. 2: 249–257. 10.1016/0022-0965(79)90087-0.

[desc70087-bib-0075] Smith, L. B. , S. S. Jones , and B. Landau . 1996. “Naming in Young Children: A Dumb Attentional Mechanism?” Cognition 60, no. 2: 143–171. 10.1016/0010-0277(96)00709-3. 8811743

[desc70087-bib-0076] Smith, L. B. , S. S. Jones , B. Landau , L. Gershkoff‐Stowe , and L. Samuelson . 2002. “Object Name Learning Provides On‐the‐Job Training for Attention.” Psychological Science 13, no. 1: 13–19. 10.1111/1467-9280.00403. 11892773

[desc70087-bib-0077] Smith, L. B. , and L. Samuelson . 2006. “An Attentional Learning Account of the Shape Bias: Reply to Cimpian and Markman (2005) and Booth, Waxman, and Huang (2005).” Developmental Psychology 42, no. 6: 1339–1343. 10.1037/0012-1649.42.6.1339. 17087565

[desc70087-bib-0078] Smith, L. , and C. Yu . 2008. “Infants Rapidly Learn Word‐Referent Mappings via Cross‐Situational Statistics.” Cognition 106, no. 3: 1558–1568. 10.1016/j.cognition.2007.06.010. 17692305 PMC2271000

[desc70087-bib-0079] Unger, L. , H. Yim , O. Savic , S. Dennis , and V. M. Sloutsky . 2023. “No Frills: Simple Regularities in Language Can Go a Long Way in the Development of Word Knowledge.” Developmental Science 26, no. 4: e13373. 10.1111/desc.13373. 36680539 PMC10293086

[desc70087-bib-0080] Welder, A. N. , and S. A. Graham . 2001. “The Influence of Shape Similarity and Shared Labels on Infants' Inductive Inferences About Nonobvious Object Properties.” Child Development 72, no. 6: 1653–1673. 10.1111/1467-8624.00371. 11768138

[desc70087-bib-0081] Willits, J. A. , E. H. Wojcik , M. S. Seidenberg , and J. R. Saffran . 2013. “Toddlers Activate Lexical Semantic Knowledge in the Absence of Visual Referents: Evidence From Auditory Priming.” Infancy 18, no. 6: 1053–1075. 10.1111/infa.12026. PMC3883433 24409090

[desc70087-bib-0082] Wojcik, E. H. , M. Zettersten , and V. L. Benitez . 2022. “The Map Trap: Why and How Word Learning Research Should Move Beyond Mapping.” WIREs Cognitive Science 13, no. 4: e1596. 10.1002/wcs.1596. 35507459

[desc70087-bib-0083] Yu, C. , Y. Zhang , L. K. Slone , and L. B. Smith . 2021. “The Infant'S View Redefines the Problem of Referential Uncertainty in Early Word Learning.” Proceedings of the National Academy of Sciences 118, no. 52, e2107019118. 10.1073/pnas.2107019118. PMC8719889 34933998

